# Biomaterial-Based bFGF Delivery for Nerve Repair

**DOI:** 10.1155/2023/8003821

**Published:** 2023-04-10

**Authors:** Qinying Huang, Bo Liu, Wencan Wu

**Affiliations:** ^1^State Key Laboratory of Ophthalmology, Optometry and Vision Science, Wenzhou Medical University, Wenzhou, China; ^2^The Eye Hospital, School of Ophthalmology & Optometry, Wenzhou Medical University, China

## Abstract

Diseases in the nervous system are common in the human body. People have to suffer a great burden due to huge economic costs and poor prognosis of the diseases. Many treatment modalities are now available that can make recovery better. Managing nutritional factors is also helpful for such diseases. The basic fibroblast growth factor (bFGF) is one of the major nutritional factors, which plays a crucial role in organogenesis and tissue homeostasis. It plays a role in cell proliferation, migration, and differentiation, thereby regulating angiogenesis and wound healing and repair of the muscle, bone, and nerve. The study on how to improve the stability of bFGF to increase the treatment effect for different diseases has garnered tremendous attention. Biomaterials are the popular methods to improve the stability of bFGF because they are safe for the living body as they are biocompatible. Biomaterials can be loaded with bFGF and delivered locally to achieve the goal of sustained bFGF release. In the present review, we report different types of biomaterials that are used for bFGF delivery for nerve repair and briefly report how the introduced bFGF can function in the nervous system. We aim to provide summative guidance for future studies about nerve injury using bFGF.

## 1. Introduction

Nerve injuries can impair sensory and motor functions in the human body, especially in the case of traumatic brain injury and spinal cord injury (SCI). A previous study has reported that 1.2 million people in the USA suffer from SCI. Some of them left the sequel [1]. The regenerative capacity of the central nervous system is limited because of its low intrinsic ability and the presence of inhibitory extrinsic factors [2]. Besides, peripheral nerve injuries can lead to local numbness and loss of function. Therefore, it is important to study how nerve repair can be promoted. Previous studies have reported that neurotrophic factors are beneficial for improving the prognosis of nerve injuries in patients. Fibroblast growth factors (FGFs) have also been studied [3–5].

FGFs are a family of proteins that have a homologous central core of 140 amino acids, with 6 identical and 28 highly conserved amino acid residues [6]. At the cellular level, FGFs regulate cell proliferation, migration, differentiation, and metabolism. They play a role in the differentiation of the inner cell mass into epiblast and primitive endoderm during the early stage of development. Macroscopically, FGFs affect organogenesis, and they respond to tissue injury [7, 8]. A total of 23 members (FGF 1–23; FGF19 of humans and chicks is the orthologue of mouse FGF15) are present in the FGFs family [9]. Basic fibroblast growth factor (bFGF), also known as FGF2, is a single-chain polypeptide with 154 amino acids, and the molecular weight of human bFGF is 18 kDa. It is a main member studied in the nervous system. bFGF was first isolated from the bovine pituitary gland, and recombinant bFGF was first reported in 1988. A previous study reported that bFGF has strong sequence conservation in different organisms. The human bFGF gene encodes 4 polypeptides with different isoforms, and it can be secreted by different types of cells [10, 11]. However, the small amount and difficult isolation of bFGF from cells cannot satisfy its needs in treatment of diseases. Researchers have explored various host, such as *Pichia pastoris*, *Bacillus subtilis*, and *Escherichia coli*, for producing abundant recombinant bFGF. At present, commercial bFGF is mainly produced and isolated by genetically modified *Escherichia coli* [12–15]. Additionally, application of bFGF gene-modified pluripotent stem cells locally can produce more bFGF to promote repair after nerve injury [16].

When bFGF binds to tyrosine kinase FGF receptors (FGFRs), the downstream signal pathways of RAS/mitogen-activated protein kinase (MAPK) and phosphatidylinositol-3-kinase (PI3K)/AKT are activated [17]. The intracellular signaling cascades of bFGF play a crucial role in normal development and also in the presence of any damage. Therefore, bFGF has many roles; it has garnered increasing attention for the studies on wound healing and tissue engineering constructs.

Biomaterials are popular substances in tissue engineering. They are substances or combinations of substances, other than drugs, synthetic or natural in origin, which can be used for any period. They can augment or replace any tissue, organ, or function of the body, partially or completely to maintain or improve the quality of life. The above concept of biomaterials was proposed during the Consensus Conference of Chester, the UK, in 1991 and was widely accepted [18]. Biomaterials are also used for tissue repair. They can be classified into natural biomaterials and synthetic biomaterials. Polysaccharides and peptides, such as chitosan and collagen, are common raw materials that are used as natural materials. If these materials are used in the living body, they can be degraded by endogenous enzymes. Therefore, these natural materials have high biocompatibility. Unlike natural materials, synthetic polymers can be designed in a specific structure to modify cell functions [19]. Some of the common requirements for a new type of biomaterial applied in tissue regeneration are as follows: biocompatibility, biodegradability, topographical and chemical surface modification, specific scaffold architecture, and proper mechanical and handling features [20]. Therefore, appropriate biomaterials are required for tissue repair in some cases of injury.

In the past decades, new methods of local drugs or protein delivery to treat diseases have been found. Researchers have found that the application of biomaterials is an excellent method to improve the curative effect. As mentioned before, bFGF is essential for many types of tissue repair. However, bFGF is unstable when it is used for nerve repair, which makes nerve repair challenging. Consequently, in the present review, we aimed to find reliable biomaterials for bFGF delivery after injury in the different nervous systems on the basis of the results of previous studies. We have also discussed some methods and mechanisms for detecting nerve regeneration. Our conclusion will be helpful for future research on nerve repair using bFGF.

## 2. Biomaterial-Based bFGF Delivery in the Different Nervous Systems

### 2.1. Central Nervous System (CNS)

CNS includes the brain, spinal cord, and optic nerve. While receiving the afferent information from the whole body, CNS becomes a coordinated motor efferent after the integration and procession of the information. Learning and memory storage are higher functions of the brain. Any injury in CNS can damage related functions. Up to now, biomaterial-based bFGF delivery has been used for nerve repair in CNS.

#### 2.1.1. Spinal Cord

SCI in CNS is the research focus to determine the strategies for nerve repair. Animal models with SCI are easier to establish, and the effects of intervention treatment can be observed easily. The role of bFGF in SCI was studied in the 1980s. A previous study reported that bFGF had a neurotrophic effect on the neurons of the spinal cord and promoted the survival of neurons [21]. bFGF was injected in the subarachnoid space or directly into the spinal cord of the SCI model during the early research stage. However, the effect of a one-shot injection was not distinct. Multiple attempts of bFGF injection aggravated the wounds of the animal models. Therefore, biomaterial-based bFGF that can release bFGF for a long time was developed to improve the therapeutic effect of bFGF.

Rabchevsky et al. [22] proposed that osmotic minipumps, containing a different dosage of bFGF, can be implanted in the lumbar thecal sac and lateral ventricle after SCI in rats, separately. This was one of the earliest studies of biomaterial-based bFGF in the treatment of SCI. bFGF was released continuously for one week from the minipumps. A similar study reported that bFGF released from the Alzet minipump was effective to reduce the injured area and preserve the zone partially in the SCI model [23]. Unlike the above studies, some studies modified the bFGF gene to improve the treatment effect. Neural stem cells (NSCs) have a limited effect on spinal cord reconstruction because the survival of NSCs is less after NSC implantation in the spinal cord. Amniotic epithelial cells (AECs) can not only increase the survival of NSCs but also promote their neural differentiation by secreting growth factors [24, 25]. bFGF gene-modified AECs increase the expression of bFGF and the survival rate of NSCs. Assuredly, cotransplantation of NSCs and bFGF gene-modified AECs improved locomotor recovery [26]. In another research, the bFGF gene was inserted in the bone marrow-derived mesenchymal stem cell (BMSC) genome which was performed later. The Basso-Beattie-Bresnahan (BBB) scores and expression of bFGF, and myelin basic protein, and NF200, were tested in rat models. The results showed that bFGF gene-modified BMSCs can modify bFGF protein successfully, which then promoted nerve regeneration and functional recovery [16]. To solve the ischemic and hypoxia problems is a potential way to repair SCI. Five hypoxia-responsive elements (5HRE) can target hypoxia loci. Therefore, researchers used lentiviral vectors (LV) to generate embryonic NSCs expressing bFGF under the regulation of 5HRE (LV-5HRE-bFGF-NSCs) [27]. The therapeutic time point, 7 days after SCI, was considered a suitable time to promote nerve repair better [28]. They found that LV-5HRE-bFGF-NSCs can target the hypoxic microenvironment in the SCI model, and the recovery of neural function was increased [27]. Another vector, adeno-associated virus 2 (AAV2), also has the characteristic of neurotropism. A recent study have shown that AAV2-5HRE-bFGF-NSCs have good therapeutic effects on SCI in rats by modulating hypoxia environment [29]. In brief, NSCs, AECs, and BMSCs are cells that can secret trophic factors automatically. When combined with bFGF, their functions in promoting nerve recovery after injury was improved via regulation of autophagy mechanism. Thus, the role of autophagy in SCI and its recovery is also worth studying.

Moreover, scaffolds and hydrogels made from biomaterials have been developed to deliver bFGF. A type of collagen scaffold (CS) made from bovine aponeurosis was used in axon regeneration after SCI. The combination between the collagen-binding domain (CBD) and bFGF can limit the fast diffusion of bFGF. CS was modified with CBD-bFGF. Compared with the control groups, the group with CS/CBD-bFGF had the best nerve recovery [30]. In another study, a type of sodium hyaluronate CS was designed. bFGF was loaded on the scaffold to form the controlled releasing system. bFGF could be released for more than 6 weeks in the SCI model [31]. This was an amazing achievement but the studies on this have not stopped yet. Lan et al. [32] prepared gelatin microspheres (GMSs) as a delivery system. bFGF was encapsulated in GMSs to promote nerve repair after SCI. They found that hydroxyl ethyl methacrylate [2-(methacryloyloxy)ethyl] trimethylammonium chloride (HEMA-MOETACL) is a biologically inert material. The positive electric charge character of HEMA-MOETACL hydrogel was beneficial to add bFGF through polyion complexation. Acellular vascular matrix and heparin (HeP) were added to the hydrogel to improve the stability of bFGF. The bFGF-HEMA-MOETACL hydrogel was then used for SCI. Examinations, such as BBB, electrophysiological studies, and histological analysis, showed that bFGF-HEMA-MOETACL hydrogel can be used to repair nerve injury [33]. Extracellular matrix has good biocompatibility because it is completely made from biological substances. Besides, the extracellular matrix is easier to combine with GF. The acellular spinal cord (ASC) is a type of acellular matrix which is made from the spinal cord after decellularization. In the SCI model, ASC was used as a part of the drug delivery system. The affinity between bFGF and ASC is strong because of the electrostatic attractions and receptor-ligand shape recognition. HeP-modified poloxamer polymer was dissolved in the bFGF-ASC solution to prepare bFGF-ASC-HP hydrogels. The time of bFGF release in bFGF-ASC-HP hydrogels was longer than that in bFGF-HeP hydrogels in vivo. Previous studies showed that bFGF-ASC-HP hydrogels not only increased neuronal survival but also promoted axonal regeneration [34, 35]. A new type of micromaterial named poly (3, 4-ethylenedioxythiophene)-coated carbon microfibers which were modified with polylysine/HeP/bFGF/fibronectin had excellent conductivity and stability. It served as a scaffold bridge to connect the injury area and then promoted axonal extension in the spinal cord [36].

Various biomaterials have been designed for bFGF delivery in the SCI model. However, the repair function is still less. Therefore, some researchers aimed to determine the effect of the combination of different trophic factors and bFGF by using a new biomaterial. Alginate scaffold, loaded with bFGF and epidermal growth factor (EGF), was prepared by mixing partial calcium-cross-linked alginate, bFGF, and EGF alginate-sulfate bioconjugates. Anterograde corticospinal tract axon tracing was used to determine the regenerative axons, which showed that regenerative axons were promoted [37]. With the development of technology, new methods have also been used to synthesize biomaterials. Microspheres of poly-lactic-co-glycolic acid (PLGA), made from glycolic acid and lactic acid, were prepared by the electrospray technique. The same method was used to encapsulate bFGF, angiopoietin-1, and vascular endothelial growth factor on PLGA. The encapsulation efficiency of angiogenic factors was higher than that of traditional methods. PLGA has good biodegradability and biocompatibility in vivo. The release assays showed that PLGA microspheres released factors for more than 2 months in vitro, and the factors remained bioactive. This was considered a further successful achievement. Diffusion tensor imaging (DTI) was a new technique to determine spinal cord recovery in this study [38]. Examination of DTI included many items such as apparent diffusion coefficient, fractional anisotropy, and diffusion tensor tractography. DTI was a potential technique to determine the prognosis of SCI repair [39]. Heparin-poloxamer (HP) hydrogel, which was loaded with bFGF and nerve growth factor (NGF), was another type of biomaterial. Poloxamer was synthesized from amphiphilic copolymers that contained units of polypropylene oxide and ethylene oxide. It increased neuronal survival and promoted axon regeneration in SCI animals [40, 41]. Delivery of bFGF along with dental pulp stem cells (DPSCs) is also a way to promote nerve repair. DPSCs are located in the dental pulp chamber. Because DPSCs inhibit the secretion of proinflammatory factors and prevent macrophages transform into M1 types, they act as a crucial regulator during nerve regeneration [42]. In a recent study, researchers encapsulated bFGF and DPSCs in HP hydrogel. The hydrogel was injected into the spinal cord after SCI. The results showed a considerable progression in nerve repair [43]. Poloxamer hydrogel and HeP hydrogel were then synthesized. High, organized growth of DPSCs was observed in HeP-bFGF hydrogel. Thus, HeP-bFGF hydrogel containing DPSCs was used for spinal cord repair. A study on the mechanism of HeP-bFGF hydrogel showed positive effects on axon regeneration by inhibiting the NF-*κ*B pathway [44]. Except for autophagy and NF-*κ*B pathway, more and more potential underlying molecular mechanisms are involved in SCI, which will be found in future studies.

To summarize, regeneration of the spinal cord is challenging, even though different biomaterials are available for bFGF delivery. An overview of the above methods for bFGF delivery is shown in Supplementary table 1.

#### 2.1.2. Brain

Diseases of the brain, such as cerebral ischemia and cerebral trauma, have a poor prognosis. bFGF is a well-known and essential neurotrophin that is present in various regions of the embryonic brain. It is an important factor to prevent neuronal death and also promotes neuronal repair [45]. Injecting bFGF in the vein or encephalocele for the treatment of brain injury is limited; therefore, drug delivery systems that can improve the functions of bFGF in the brain were developed gradually.

Parkinson's disease (PD) and Alzheimer's disease (AD) are common diseases in older people. bFGF can promote the recovery of patients with PD or AD. Early studies focused on using bFGF to improve the survival of grafted neurons in transplantation. Implanting fetal ventral mesencephalic grafts was one of the methods used for PD treatment. The disadvantage of the method was the limited outgrowth of grafts in the host striatum. The grafts along with bFGF showed better outgrowth, and the innervation density increased significantly [46]. Decellularized brain extracellular matrix (dcBECM) is found in the brain. It has a three-dimensional ultrastructure and contains nutritional components. When dcBECM was used as a novel vehicle, it could deliver drugs to a specific site of the body because it had good biocompatibility. In patients with PD, a mixture of bFGF and dcBECM injection could release bFGF for a long time [47]. The role of bFGF should also be studied in AD. To alleviate the side effects of systemic medication, bFGF nasal spray was designed for the treatment of AD. Chitosan was added to the spray to increase the permeation of bFGF, and HeP was used to increase the stability of bFGF. The uptake of bFGF in brain in the nasal spray group was more than in the groups that used intravenous or intranasal administration. Besides, bFGF nasal spray significantly improved spatial memory impairment by alleviating neuronal degeneration in rat hippocampus [48]. The biomaterial can also be made using the nanoparticles of polyethylene glycol (PEG) and PLGA. When PEG-PLGA was modified with lectins, it could increase the drug absorption in the brain via the intranasal pathway. bFGF was encapsulated in the modified nanoparticles and was then delivered to the brain following intranasal administration in an AD model of rats. The radioisotopic tracing method was used to determine bFGF distribution in the brain. The result showed that the areas of bFGF distribution and spatial learning and memory were both increased [49]. Moreover, intranasal delivery of bFGF was also applied in the stroke model of rats. bFGF-nanoliposome system was made to bypass the blood-brain barrier. The PI3-K/AKT pathway was activated by bFGF, thereby involved in brain recovery [50]. It seemed that drug delivery through intranasal way was a noninvasive and practical method for treating many brain diseases, especially AD. More drug research should focus on this method to improve treatment effects.

The efficiency of drug delivery to the brain is mainly affected by the blood-brain barrier. Thus, the delivery systems that can cross the blood-brain barrier can be helpful. Yemisci et al. [51] designed a type of chitosan-polymers nanoparticles that targeted transferrin receptor-1 on brain endothelia. When bFGF was loaded in nanoparticles, it could cross the blood-brain barrier via receptor-mediated transcytosis. Another type of nanohybrid hydrogel, made from polyelectrolyte complex nanoparticles and sulfated glycosaminoglycan, had similar properties as the brain extracellular matrix. bFGF and stromal-derived factor-1 *α* were stored in the hydrogel via electrostatic sequestration. The controlled release of bioactive factors could recruit endogenous NSCs and regulated their fate. The factors increased angiogenesis and neurogenesis in the ischemic stroke model to promote functional recovery [52].

As discussed before, genetic engineering can also be used for bFGF delivery. Research performed by Fujiwara et al. established a bFGF-secreting cell line using baby hamster kidney (BHK) cells. bFGF gene was inserted in the BHK cell genome via genetic manipulation. The graft of bFGF-BHK cells was transplanted into the brain of an ischemic injury rat model [53]. NSCs have large differentiative potentials such as differentiating to neurons and astrocytes. Therefore, NSC transplantation was also applied for stroke repair [54]. bFGF gene-modified NSCs can be more useful in nerve recovery because of its neurotrophic effect. Indeed, the proliferative and differentiative capabilities of NSCs were improved after bFGF gene-modified NSC implantation in the rat. Similarly, a reduction of infarct volume in the brain was observed, although no corresponding improvement was found in neurological function [55]. More studies should be performed to explain this phenomenon.

Brain recovery after injury is very complex. Presently, many drugs used for brain treatment are delivered in the blood vessels. However, the blood-brain barrier is a crucial factor that weakens the effect of treatment. Besides, some drugs fail to reach the brain because they are the first metabolism in the liver. As shown above, different biomaterial-based bFGF delivery pathways or bFGF genetic engineering can be used; however, they are now not sufficient for ideal recovery.

#### 2.1.3. Optic Nerve

The optic nerves are a part of the CNS. Ocular trauma or tumors can lead to optic nerve injury. Optic nerve damage was recognized irreversibly in the past. However, increasing evidence shows that the optic nerve can regenerate partially after injury. The promotion of intrinsic regeneration and reduction of extrinsic inhibited factors are significant. Supplement exogenous nutrition factors are also useful [56]. bFGF, as one of the neurotrophic factors, can promote optic nerve repair.

Early studies suggested that bFGF injection into the eyeball did not protect the survival of retinal ganglion cells (RGCs). Nevertheless, RGC protection was detected when bFGF was injected at the site of optic nerve injury [57, 58]. Xie and team [59] performed a study on rats, where AECs were modified with the human bFGF gene. AECs were injected at the site of optic nerve injury in rats. RGC survival and growth-associated protein 43 (marker of regenerative axons) levels in the AEC group were higher than those in the group without AEC injection. Studies on biomaterial-based bFGF delivery in optic nerve repair after injury are scarce. Hence, new types of biomaterials should be used to deliver bFGF tentatively.

### 2.2. Peripheral Nervous System (PNS)

Anatomically, the PNS consists of cranial, spinal, and autonomic nerves. It acts as a bridge to link peripheral receptors to the CNS. Therefore, the PNS is also a key part of the nervous system. Autologous nerve transplantation is the gold standard for repairing peripheral nerve injury although the PNS has a partial regenerative capacity [60]. However, autografts will cause damage to normal tissues. The combination of biomaterials and bFGF can promote peripheral nerve regeneration.

#### 2.2.1. Sciatic Nerve

Sciatic nerve models are often used to explore nerve repair methods after injury in PNS injury. As the transverse injury is frequent in the PNS, nerve tubes are commonly used to connect nerve stumps. In an early study, a transplanted tube, synthesized with a polymer, was loaded with bFGF and alpha-1 glycoprotein. The outer surface of the tube was coated with an impermeable polymer to release bFGF only in the tube lumen. Further, the tube was transplanted to the location of the resected nerve after an 8 mm segment of the sciatic nerve was removed. Followed by bFGF linear release, both myelinated and unmyelinated axons of the sciatic nerve were regenerated in the tube [61]. In another study, a nerve guide with a two-ply structure (dense inner layer and microporous outer layer) was designed. It was composed of poly (D, L-lactide), and bFGF was embedded in the inner layer to ensure bFGF release in the guide lumen. The results obtained using a rat model showed that Schwann cell proliferation was accelerated, and the transected 15 mm sciatic nerve was entirely regenerated [62]. Furthermore, regenerative nerve functions should be tested in detail although recovery was observed.

The application of hydrogel or a hydrogel-related nerve duct is also suitable for bFGF delivery. Hence, bFGF was impregnated in gelatin hydrogel, which was applied around the sutured allografts of the sciatic nerve. Researchers found that sciatic nerve segments from dogs regenerated axons when transplanted to common peroneal nerves in the hind limb of other dogs [63]. A new type of gel consisting of HeP and alginate was obtained by crosslinking with ethylenediamine. The hydrogel, encapsulated with bFGF, was added to the site where a small segment of the sciatic nerve was resected. The diameter and numbers of regenerated axons increased with increasing bFGF release. Vascularization was also observed [64]. New blood vessels might have promoted nerve regeneration via nutrient transportation. Later, a hollow chitosan conduit was prepared for bFGF delivery. bFGF was mixed in fibrin-based gel, and the inner surface of the conduit was coated with the gel. In rats, the regenerative function of the sciatic nerve was the same between the autograft and chitosan conduit filled with the bFGF-delivery gel. The efficiency of bFGF release could be assessed by measuring the remaining radioactivity when bFGF in the gel was labeled with iodine-125 [65]. bFGF-PLGA microspheres, another type of biomaterial, could be injected into a silicone tube after the tube was sutured in the defected area of the sciatic nerve [66]. Biodegradable materials such as nerbridge and polyglycolic acid were used for preparing the nerve conduit. bFGF-GMSs or bFGF with the polycystic kidney disease domain and CBD were injected inside the conduit to regenerate axons [67, 68]. Most recently, a new type of engineered biomaterial was designed. bFGF was firstly loaded with chitosan particles, and then, the complex was added to the type I collagen solution. The chitosan particles were injected into the chitosan conduits before using them in a more challenging nerve injury model, where 20 mm of the sciatic nerve was excised. A cholera toxin B tracing experiment was performed to assess whether the regenerative nerve had crossed the defective area. The result showed a good treatment effect [69]. The study provided evidence that biomaterial-based bFGF delivery could be used in severe sciatic nerve injury.

A nerve duct with an ordered arrangement is beneficial for promoting nerve growth. Bovine aponeurosis was originally used for making linear ordered collagen scaffold (LOCS) fibers. LOCS was put inside a collagen tube after combining it with CBD-bFGF. As mentioned before, the CBD was a specific structure that contributed to continuous bFGF release. The tube was sutured at the defected site of the sciatic nerve. Apart from conventional examinations, fluorogold retrograde tracing and gastrocnemius muscle examination were performed to verify the axon regenerative effect [70]. In another study, pepsin-solubilized porcine skin type-I atelocollagen was mixed with acetic acid to obtain oriented collagen tubes (OCTs) at a specific condition. In rats, the OCTs were put into a solution containing bFGF to be directly loaded with bFGF, which further accelerated nerve repair [71]. Directional tubes promoted regenerative nerves to grow in the same direction as that of the original nerve.

As shown in the CNS, bFGF delivery with stem cells or other neurotrophic factors also promotes peripheral nerve repair. Induced pluripotent stem cells (iPSc) played a supportive functional role for cells. Researchers developed a bioabsorbable polymer tube, which was combined with bFGF and iPSc. The tube consisted of two layers. Poly L-lactide (PLA) multifilament fiber was used to synthesize the outer layer, whereas 50% poly e-caprolactone porous sponge and 50% PLA formed the inner layer. The inner layer of nerve conduits was loaded with iPSc neurospheres and biodegradable GMSs with bFGF. The study results found that the nerve conduits loaded with iPSc and bFGF promoted sciatic nerve regeneration better in mice [72]. Besides SCI, the HP hydrogel was also applied in diabetic rats with a sciatic nerve crush injury. bFGF and NGF were both encapsulated in the HP hydrogel. Compared with other experimental groups, the group of HP hydrogel codelivered with GF had a stronger protective function. Molecular pathways such as mitogen-activated protein kinase (MAPK)/extracellular-signal-regulated kinase (ERK), phosphoinositide 3-kinases (PI3K)/AKT, and Janus kinase JAK/signal transducer and activator of transcription 3 (STAT3) were activated to develop a positive effect [73]. Similarly, the autologous vein promoted nerve recovery by wrapping sciatic nerve injury. As the autologous vein was limited, a collagen sheet impregnated with bFGF was developed. The result of nerve repair between the collagen sheet and vein was comparable [74, 75]. Recently, a new type of nerve conduit which was composed of polylactide and polycaprolactone was prepared for sciatic nerve repair in a mice model. Stromal cell-derived factor-1 (SDF-1), a chemokine, recruited stem cells. Gelatin hydrogel microspheres that incorporated bFGF or SDF-1 were made with the glutaraldehyde cross-linking of acidic gelatin. The mixture of bFGF gelatin and SDF-1 gelatin was injected into the nerve conduit. The result showed that the continuous release of bFGF and SDF-1 effectively promoted nerve repair [76]. To conclude, the delivery of biomaterial-based bFGF and other trophic factors is a popular method to repair nerve injury.

Various biomaterials have been prepared to repair nerve injury in the sciatic nerve. Most of them are suitable for bFGF delivery. The overview of biomaterials used for bFGF delivery in sciatic nerve injury models is shown in Supplementary table 2.

#### 2.2.2. Facial Nerve

A facial nerve injury model is another common model for exploring methods of nerve repair in the PNS. An osmotic minipump is a classical way to continuously release bFGF. In a study, bFGF was combined with HeP before loading the complex in the minipump. The facial nerve was repaired with a primary epineural suture after partial transection in a rat model. Then, the pump was implanted subcutaneously. bFGF was released to the incision area of the nerve via a tube. The results showed that bFGF increased the number of nerve fibers and promoted nerve function recovery [77]. Komobuchi et al. [78] designed a new type of hydrogel by cross-linking gelatin with glutaraldehyde. The gelatin originated from bovine bone collagen. In another facial nerve injury model of pig, a hydrogel loaded with bFGF was added. The hydrogel group showed better results than other groups without bFGF or the one-shot application of bFGF in the assessment of facial movements and electrophysiological testing. The number of regenerative nerve fibers was also more than the other groups. Clinically, bFGF was mixed with the gelatin hydrogel, and then, the gel was applied to the exposed facial nerve after decompression surgery in patients with Bell palsy. The recovery rates in the bFGF group were higher than those in the traditional group [79]. It was a rare study where the application of biomaterial-based bFGF was tested in patients.

Most researchers focus on developing nerve tubes to promote nerve repair. A minipig experimental model was used to observe facial nerve regeneration. The facial nerve of the minipig is long enough to be operated on and has similar anatomy to that of the human. In a study, a 35 mm facial nerve, a longer peripheral nerve defect, was replaced with a collagen nerve conduit. The conduit was loaded with bFGF or ciliary neurotrophic factor (CNTF). The results of electrophysiology evaluation and histological analysis showed that the treatment with bFGF and CNTF was better than the treatment with only bFGF or CNTF for nerve regeneration [80]. GMSs containing bFGF were added inside a silicone tube. The experiments on facial nerve injury in a rat model showed that the sustainable release of bFGF in the early stage promoted nerve repair [81]. Besides, a collagen conduit was combined with bFGF to repair facial nerve injury in rabbits. The results of the electrophysiological examination and histological examination showed that the collagen conduit with CBD-bFGF was helpful for nerve repair [82].

As said before, the application of neural stem/progenitor cells (NS/PCs) is effective for nerve repair. In a rat model with a facial nerve defect, a nerve conduit made from rat-tail collagen and HeP was implanted. NS/PCs were labeled with Hoechst and loaded into the nerve conduit. bFGF was also loaded in the tube, which promoted NS/PC survival. The labeled NS/PCs could be observed even 3 months after the implantation [83]. Thus, stem cell implantation with bFGF plays an important role in repairing nerve injury.

#### 2.2.3. Other Peripheral Nerves

The HeP/alginate gel was used to repair a thumb nerve injury in a patient. bFGF was loaded in the gel, and the gel was applied to the suture area of the broken finger. Tinel's sign appeared again after surgery. Both moving and static 2-point discrimination functions in the thumb recovered partially [84]. The study showed that the delivery of biomaterial-based bFGF was feasible to repair limb nerve injury.

## 3. Discussion

In our review, we conclude different biomaterials that are used for releasing bFGF for nerve repair. The nerve regenerative function in the CNS is poorer than that in the PNS. As mentioned before, many factors limit CNS regeneration after injury. The CNS gradually lost its intrinsic growth during the developmental process because of changes in gene expression. Krüppel-like factors (Klf) are a series of transcription factors important for the regulation of CNS regeneration. In vitro, the overexpression of Klf-4 and Klf-9 inhibited axon growth in neurons, whereas the positive regulators Klf-6 and Klf-7 promoted axon growth. Inhibition of regulatory pathways such as PI3K/AKT and JAK/STAT3 pathways was observed in the CNS, leading to low growth capacity. The different environments after injury in the CNS and PNS also led to differences in their repair functions. In the CNS, myelin-related molecules, including oligodendrocyte-myelin glycoprotein, myelin-associated glycoprotein, and isoforms of the reticulon-like protein Nogo, were considered major factors that inhibited nerve regeneration. However, the grafts from the PNS implanted in the CNS promoted neural growth. Cytokines secreted by Schwann cells played a role in nerve regeneration. Furthermore, inflammatory and trophic factors acted as regulators. bFGF is an environmental factor that promotes nerve regeneration.

**Figure 1:**
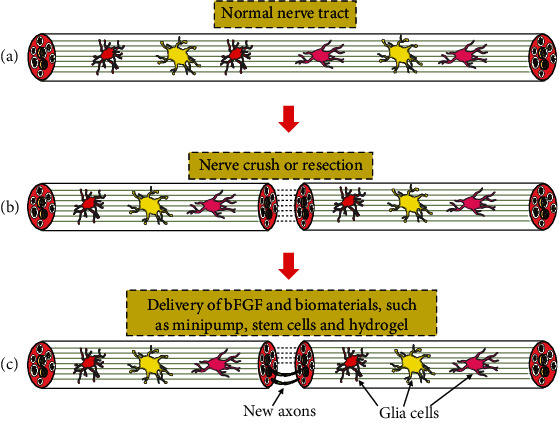
Normal nerve tract and nerve repair process. (a) Normal nerve tract and different kinds of glia cells, including microglia, astrocytes, and oligodendrocytes, are well-ordered inside the nerve tract. (b) The nerve tract is injured after exposing to the external force. The broken line means nerve crush or resection. Next, the morphology and function of glia cells will undergo a series of changes. (c) bFGF is delivered to the local injury area by biomaterials. The green full line means regenerative nerve. The glia cells also act an important role during the repair process.

As nerve injury is common, any effort to improve its outcome should be encouraged. Based on the aforementioned studies, bFGF delivery systems mainly include a minipump, hydrogel, nerve conduit, and the combination of grafts and bFGF both in the CNS and PNS. In addition, the technique by which the bFGF gene is inserted into stem-cell genomes using genetic engineering is proven effective. Particularly, the application of bFGF with other neurotrophic factors is an essential way. All methods can achieve the goal that bFGF is delivered locally and continuously. Some of biomaterials that are used in the CNS can also be used in the PNS. The experimental results showed better nerve recovery function of nerve in groups with biomaterial-based bFGF delivery (Figure 1). Some biomaterials are never used in a nerve injury model but promote nerve growth in vitro. Nanoparticles made from chitosan and fucoidan were formed by electrostatic interactions. Fucoidan limited the rapid release of bFGF as HeP did. The nanoparticles were put into a medium with PC12 cells after loading with bFGF. Growth effects but no cytotoxic effects on the cells were observed [85]. Similarly, a bFGF delivery system was made from HeP and polyethylene arginylaspartate diglyceride. PC12 and SH-SY5Y cells were used to test the suitability of the system. The group containing bFGF and NGF promoted cell growth and proliferation better than those containing only bFGF or NGF [86].

**Figure 2:**
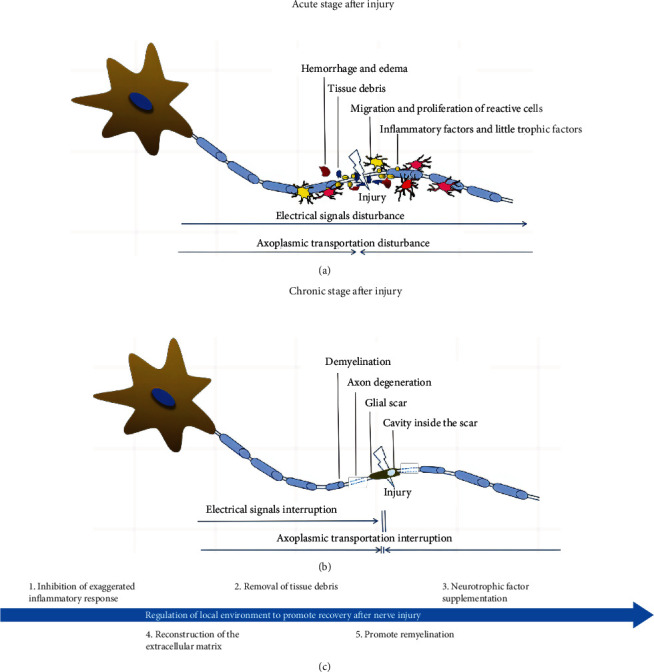
Pathological changes in nerve tract after injury and strategies for neural repair. (a) The nerve tract responds immediately and acutely when it is damaged. The acute reactions include hemorrhage, edema, and inflammation. The function of neural electrical transmission is impaired. (b) When the damage comes to chronic stage, pathological changes such as demyelination and axon degeneration will appear locally, leading to the electrical signal interruption. (c) There are several strategies for nerve repair that can be followed after nerve injury.

How does bFGF promote neuron survival and axon regeneration after a nerve injury? We have to learn about the distribution of FGFRs in nervous system firstly. There are four types of FGFRs. FGFR1-3 are abundantly expressed in the nervous system but FGFR4 is hardly expressed in adult brain. Furthermore, FGFR1 is mainly expressed in neurons although it is also expressed in glia cells. FGFR2-3 have abundant expression in astrocytes and oligodendrocytes while FGFR1 and FGFR3 are expressed in microglia except FGFR2 [87–89]. Normally, bFGF can bind with FGFR1-3, then promoting brain growth and development, which is also related to learning, memory, and other advanced brain functions. Upon pathological condition, the secretion of bFGF mainly by neurons and astrocytes is increased, and bFGF continues to act on neurons and glia cells to promote nerve repair [90–92]. Another growth factor, FGF21, is found to interact with FGFR1-2 and then regulates cellular metabolic functions. Research conducted by Zhu et al. found that FGF21 also could promote SCI recovery in the rat model although it was delivered via tail vein injection [93, 94]. When the tissue was damaged, positive pathways such as MAPK/ERK, PI3K/AKT, and JAK/STAT3 were activated by bFGF. Histologically, vascularization was activated, and inflammatory cells were inhibited. Besides, bFGF-responsive progenitor cells were more sensitive to bFGF [95]. A recent study suggested that bFGF effectively eliminated myelin debris in a sciatic-nerve-crush rat model, and autophagy was activated in neurons [96]. It also implies that bFGF affects neuronal cells and nonneuronal cells. Astrocyte proliferation was promoted by exogenous bFGF after nerve injury, but its activation was inhibited. Inflammatory factors secreted by astrocytes were decreased, and glial scar formation was also suppressed [97]. Researchers found that astrocytes infiltrated into the collagen-based hydrogels containing FGF-2 after its injection to the area of SCI. The cell would fill the sac here and promote the recovery of spinal cord function [98]. These events are beneficial for axon regeneration. Besides, astrocytes can secrete bFGF by themselves. Endogenous and exogenous bFGF affect oligodendrocyte and microglial activities. bFGF promotes the proliferation and activation of oligodendrocyte precursor cells (OPCs). OPCs differentiate into mature oligodendrocytes, helping in the remyelination of regenerative axons [99, 100]. The migration and activation of microglia are also observed in the injured area. Interestingly, bFGF promotes the transformation of the M1 inflammatory phenotype of microglia into the M2 anti-inflammatory phenotype. Microglial activation is beneficial to clear debris and relieve inflammation [89, 101, 102]. Thus, bFGF promotes nerve repair after injury by controlling the activities of various types of cells. The regenerative effect of axons can be improved if negative-regulation signals in neurons are suppressed while delivering bFGF. The endogenous inhibitory pathways, such as suppressor of cytokine signaling 3/JAK and phosphatase and tensin homolog/phosphatidylinositol 3,4,5-triphosphate, are activated after nerve injury as reported in many studies [103–106]. The specific gene knockdown is a popular way to reduce the inhibitory effect. However, gene knockdown is impossibly practiced in the human body. Hence, we can deliver other molecules along with bFGF or design a biomaterial with an active domain, aiming to combine with the receptors of interest and then block the negative signal pathways in cells. Mild axon regeneration occurs as a reactive effect in an acute stage after nerve injury, but the regenerative phenomenon disappears quickly because of the inhibited microenvironment. In the chronic stages, the glial scarring will stop axons that are extending heavily (Figure 2). Hence, it is better that the biomaterial or the other molecules along with bFGF regulate the positive and negative signals before reaching the chronic stage.

Various types of biomaterials may not be used for nerve repair. For example, a three-dimensional scaffold made from heparin-functionalized chitosan-alginate polyelectrolyte complexes were designed to deliver bFGF. bFGF was active after its release from the scaffold [107]. It is worth applying biomaterials that have not been used in the nervous system to promote nerve repair after injury. However, some characteristics of biomaterials should be considered. First, biomaterials should not have a toxic effect on cells. Biomaterials that can accelerate cell proliferation and differentiation will be better. Second, the body should not have a serious exclusionary reaction to the biomaterial, i.e., the material should coexist with the body. Third, biomaterials that can deliver bFGF over an extended period while maintaining its bioactivity will be better. HeP is often combined with bFGF to chronically release bFGF. Last, biomaterials that can be degraded gradually in the body during bFGF delivery are more suitable [108, 109]. A popular view is to develop biomaterials from a matrix in the body because the extracellular matrix has higher biocompatibility, and it is easy to combine with bFGF. Besides, the extracellular matrix can be degraded by enzymes. Large molecular biomaterials based on the extracellular matrix, such as chitosan and collagen fibers, have been tested for their applicability [110]. However, some of them have a low capacity to load with enough bFGF. Thus, more small molecular biomaterials have been developed later. Biomaterial domains or bFGF have also been modified. The packaging efficiency of bFGF has been improved. To improve the recovery effect, biomaterials containing bioactive compounds, such as insulin growth factor-1 and biotin, have also been developed [111]. The aforementioned ways have been proved to be useful.

Hydrogel is the most-preferred drug delivery system including the bFGF delivery systems as mentioned previously. It can be developed from biomaterials existing in the body. Hydrogel is a thixotropic, adhesive, and three-dimensional network polymer that can be swollen with water because of its hydrophilic groups; however, is insoluble in water. According to different classification criteria, hydrogels can be divided into different types. Based on their reaction to the external environment, hydrogels are classified into traditional hydrogels and environmentally sensitive hydrogels. Generally, temperature, ion, and PH are the main environmental factors that affect hydrogel characteristics [112, 113]. When bFGF hydrogels are under a specific condition, bFGF can be released as characteristics of hydrogels change. It is a high-potential method to promote nerve regeneration. Small molecular hydrogels encapsulate more bFGF dose. Furthermore, hydrogels can be used locally, and bFGF is released persistently. Moreover, hydrogels developed from biomaterials in the body have higher biocompatibility. In the future, based on this review and previous studies, more biomaterial hydrogels will be developed to deliver bFGF.

## 4. Conclusion

Injuries in nervous system are severe and challenging. Although there are many methods used for nerve repair, drug delivery locally is more effective. Biomaterials can serve as this kind of delivery system. It cannot be ignored that bFGF is an important neurotrophic factor which can promote nerve repair. Thus, combination of biomaterials and bFGF is a valuable way for nerve repair. New types of biomaterials will be developed in the future.

## Data Availability

The data used to support the findings of this study are included within the article.

## Supplementary Materials

Supplementary Materials Supplementary table 1: different kinds of biomaterials used for bFGF delivery in spinal cord after injury. Supplementary table 2: various of biomaterials used for bFGF delivery in sciatic nerve after injury.
