# Supplementary material for: Biomaterial-Based bFGF Delivery for Nerve Repair
Source: Oxid Med Cell Longev. 2023 Apr 10;2023:8003821. doi: 10.1155/2023/8003821 (PMC10110389; doi:10.1155/2023/8003821)
Supplement: Supplementary Materials — Supplementary table 1: different kinds of biomaterials used for bFGF delivery in spinal cord after injury. Supplementary table 2: various of biomaterials used for bFGF delivery in sciatic nerve after injury. [file 8003821.f1.doc]

**Supplementary table 1**: **Different kinds of biomaterials used for bFGF delivery in spinal cord after injury.**

| Biomaterials | Original materials | Drugs delivery | Animals | Studies |
| --- | --- | --- | --- | --- |
| Osmotic minipump | -- | bFGF | Rats | Lee *et al.*, 1999;  Rabchevsky *et al.*, 2000 |
| Co-transplantation of NSCs and bFGF gene-modified AECs | NSCs, bFGF gene-modified AECs | NSCs, bFGF | Rats | Meng *et al.*, 2008 |
| bFGF gene-modified BMSCs | -- | bFGF | Rats | Liu *et al.*, 2011 |
| LV‐5HRE‐bFGF‐NSCs,  AAV2‐5HRE‐bFGF‐NSCs | bFGF gene-modified NSCs | bFGF | Rats | Zhu *et al.*, 2020  Zhu *et al.*, 2021 |
| Collagen scaffold | -- | CBD-bFGF | Rats | Shi *et al.*, 2014 |
| HEMA-MOETACL hydrogel | HEMA, MOETACL, ethylene dimethacrylate, acellular vascular matrix | bFGF | Rats | Chen *et al.*, 2015 |
| bFGF-ASC-HeP hydrogel | ASC, HeP modified poloxamer polymer | bFGF | Rats | Xu *et al.*, 2016; Liu *et al.*, 2017 |
| PEDOT-MF | PEDOT, PLL/heparin/bFGF/fibronectin | bFGF | Rats | Alves-Sampaio *et al.*, 2016 |
| Alginate scaffold | -- | EGF, bFGF | Rats | Grulova *et al.*, 2015 |
| PLGA microspheres | Glycolic acid, lactic acid | VEGF, Ang-1, bFGF | Rats | Yu *et al.*, 2016 |
| HP hydrogel | Heparin, poloxamer | NGF, bFGF | Rats | Cortiella *et al.*, 2006; Hu *et al.*, 2020; Albashari *et al.*, 2020 |
| Co-transplantation of bFGF and DPSCs | DPSCs | DPSCs, bFGF | Rats | Luo *et al.*, 2018 |
| Sodium hyaluronate CS | Sodium hyaluronate, CS | bFGF | Rats | Shang *et al.*, 2019 |

AAV2: adeno-associated virus 2; AECs: amniotic epithelial cells; Ang-1: angiopoietin-1; ASC: acellular spinal cord; bFGF: basic fibroblast growth factor; BMSCs: bone marrow-derived mesenchymal stem cells; CBD: collagen binding domain; CS: collagen scaffold; DPSCs: dental pulp stem cells; EGF: epidermal growth factor; HEMA: hydroxyl ethyl methacrylate; HeP: heparin; HP: heparin poloxamer; LV: lentiviral vectors; MOETACL: [2-(methacryloyloxy)ethyl] trimethylammonium chloride; NGF: nerve growth factor; NSCs: neural stem cells; PEDOT-MF: poly (3, 4-ethylenedioxythiophene)-coated carbon microfibers; PLGA: poly-lactic-co-glycolic acid; PLL: polylysine; VEGF: vascular endothelial growth factor; 5HRE: five hypoxia‐responsive elements.

**Supplementary table 2:** **Various of biomaterials used for bFGF delivery in sciatic nerve after injury.**

| Biomaterials | Original materials | Drugs delivery | Animals | Studies |
| --- | --- | --- | --- | --- |
| Polymer tube with an impermeable polymer coat | Stainless  steel wire mandrel, EVA | bFGF, alpha 1-GP | Rats | Aebischer et al., 1989 |
| Nerve guide with a two-ply structure | Poly (D, L-lactide) | bFGF | Rats | Wang et al., 2003 |
| Gelatin hydrogel | Gelatin | bFGF | Dogs | Ide et al., 1998 |
| H/A hydrogel | HeP, alginate | bFGF | Rats | Ohta et al., 2004 |
| Chitosan conduit with fibrin-based hydrogel | Chitosan, fibrin | bFGF | Rats | Han et al., 2010 |
| Silicone tube with bFGF-PLGA microspheres | Silicone, PLGA | bFGF | Rats | Si et al., 2017 |
| Nerve conduit with bFGF-GMSs | Nerbridge, polyglycolic acid | PKDD-CBD-bFGF | Rats | Fukuda et al., 2018; Fujimaki et al., 2020 |
| Chitosan conduit with bFGF chitosan particles | Chitosan | bFGF | Rats | Liu et al., 2021 |
| Collagen tube with CBD-bFGF/LOCS | Collagen, LOCS | bFGF | Rats | Ma et al., 2014 |
| OCTs | Epsin-solubilized porcine skin type-I atelocollagen, nacetic acid | bFGF | Rats | Fujimaki et al., 2017 |
| Nerve conduit with iPSc neurospheres and bFGF-GMSs | PLA, PCL | bFGF | Mice | Ikeda et al., 2014 |
| HP hydrogel | Heparin, poloxamer | NGF, bFGF | Rats | Li et al., 2018 |
| Collagen sheet with bFGF | Collagen | bFGF | Rats | Mukai et al., 2019 |
| Nerve conduit with bFGF/SDF-1 gelatin | Polylactide, polycaprolactone | bFGF, SDF-1 | Mice | Shintani et al., 2020 |

alpha 1-GP: alpha-1 glycoprotein; bFGF: basic fibroblast growth factor; EVA: Ethylene-vinyl acetate copolymer; GMSs: gelatin microspheres; H/A: Heparin/alginate; HP: heparin poloxamer; LOCS: linear ordered collagen scaffold; OCTs: oriented collagen tubes; PCL: poly e-caprolactone; PKDD-CBD: polycystic kidney disease domain-collagen binding domain; PLA: poly l-lactide; PLGA: poly-lactic-co-glycolic acid; SDF-1: stromal cell-derived factor-1.
